# Supplementary material for: Learning curve and surgical outcome of robotic assisted colorectal surgery with ERAS program
Source: Sci Rep. 2022 Nov 29;12:20566. doi: 10.1038/s41598-022-24665-w (PMC9709162; doi:10.1038/s41598-022-24665-w)
Supplement: Supplementary file 2 — Supplementary Information 2. [file 41598_2022_24665_MOESM2_ESM.zip › RAL Raw data-Ñ[▒K/129/OP N_ñw╝╨░O▒Kñσ.pdf]

W52 / W52

臺中榮民總醫院
手術室護理記錄

[redacted]

基本資料

- 室別： 開刀房-17室，手術科別： CRS，手術類別： 常規，日期： 2021/11/24
- 進入時間： 1200 ； 麻醉開始時間： 1205 ； Time Out時間： 1310 ； 劃刀時間： 1311 ； 手術結束時間： 2030 送出時間： 2100 ；
- 麻醉方式： GE
  診斷：Malignant tumor of rectum
  手術名稱：Robot Assisted Low Anterior Resection
  手術局部用藥：nil
  術前預防性抗生素：
  - 抗生素名稱一： Veterin 1GM IVD由麻醉護理人員執行
  - 時間： 1300
  - 上次簽章: 朱淨慧

  ，追加預防性抗生素：
  - 抗生素名稱一： Veterin 1GM IVD由麻醉護理人員執行
  - 時間： 1700
  - 上次簽章: 張秀敏
- 手術醫師： 蔣鋒帆，林陳彥，蔡濬樟
- 麻醉醫師： VS 施涵薰
-
  - 刷手護士A ： 歐真勤，時間： 1200，刷手護士B ： 許雅芳，時間： 1230，接替刷手一： 王昱琄/劉雅汶，時間： 1500，接替刷手二： 張秀敏，時間： 1730
  - 流動護士A ： 朱淨慧，時間： 1200，接替流動一： 張秀敏，時間： 1500，接替流動二： 劉雅汶，時間： 1700
- 更多接替及流動護士： 接替流動三： 洪豐麟，時間： 1900

皮膚狀況

A-V Shunt： 無
術前： 完整
記錄者： 上次簽章: 朱淨慧
術後： 完整
記錄者： 洪豐麟

手術準備

手術臥式 單一手術臥式

-
  - 手術臥式： 截石臥式
  - 使用附件： 溫毯 38 ℃，頭圈，腳架
  - 皮膚準備： 10%Aq Betadine，Hibiscrub，2% Chlorhexidine
  - 電燒： 使用電燒: 電極片位置： 其它： 電燒迴路軟墊，雙極電燒，記錄者： 上次簽章: 朱淨慧

止血帶T： 無

植入物

- 植入物名稱/號碼/數量/部位：ethicon GIA:45-4.3*2/rectum

，記錄者：上次簽章: 洪豐麟

標本送檢

- 細菌培養 無
- 細胞學 無
- 冰凍切片 無
- 病理標本
  組織名稱 ：rectum
  ，

組織名稱二： distal cut end

,

組織名稱三： IMA Root

- 截肢 無

標本交付 標本： 3 袋，標本： 0 盒，標本： 0 瓶，細胞學： 0 管，Culture Tube： 0 管，被交付醫師： Fellow 林陳彥，

- 記錄者： 上次簽章: 洪豐麟
- 時間： 1925

傷口術後敷料

傷口敷料： 傷口敷料： 無

- 請於圖上標示部位：

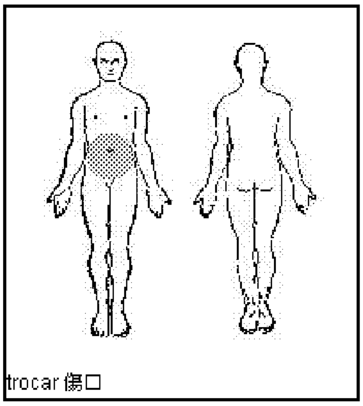

- 傷口敷料： 軟性敷料

導管

導管	天數	導管資訊	插入日期	插入時間	更換日期	拔除日期	拔除時間	拔除原因	預計拔除/更換	預計拔除/更換日期
Double Lumen CVC(1)	1	號碼:8 Fr.; 深度:15cm; 部位:jugular (右); 象限:RU;	2021/11/24	1240						
Epidural PCA(1)	1	部位:硬脊膜下;	2021/11/24	1400						
Foley(1)	1	號碼:16 Fr.; 部位:膀胱;	2021/11/24	1255						
A-line (1)	1	號碼:20 G; 部位:左手;	2021/11/24	1235						

用物計數

- 手術中出血量： minimal c.c.
- 手術中尿量： 500 c.c.
-
    - 其它： 無

用物計數： 多切口同時縫合

- 1.器械： 正確，2.縫針： 正確，3.刀片： 正確，4.紗布： 正確，5.腹部墊： 未使用，6.紗球： 未使用，7.PEANUTS ： 未使用，8.紗條： 未使用，9.絲帶： 未使用，10.針頭： 未使用，11.Vessel loop： 未使用，12.Boots： 未使用，13.Jelco： 未使用，14.棉條： 未使用，
    - 第一次：
        - 刷手護士 ： 張秀敏
        - 流動護士： 上次簽章: 劉雅汶
        - 時間： 1800

,

    - 第二次：
        - 刷手護士 ： 張秀敏
        - 流動護士： 上次簽章: 劉雅汶
        - 時間： 1820

,

    - 第三次：
        - 刷手護士 ： 張秀敏

- 流動護士： 上次簽章: 洪豐麟
- 時間： 1900

備註

備註：davinci scope No.1

- 記錄者： 上次簽章: 朱淨慧
- 時間： 1320

填寫護理人員 洪豐麟

正式摘要

W52 / W52

doctorRank:B
